# Corneal Epithelial–Stromal Fibroblast Constructs to Study Cell–Cell Communication in Vitro

**DOI:** 10.3390/bioengineering6040110

**Published:** 2019-12-04

**Authors:** Tina B. McKay, Dimitrios Karamichos, Audrey E. K. Hutcheon, Xiaoqing Guo, James D. Zieske

**Affiliations:** 1 Schepens Eye Research Institute/Massachusetts Eye and Ear, Department of Ophthalmology, Harvard Medical School, Boston, MA 02114, USA; Tina_McKay@meei.harvard.edu (T.B.M.); Audrey_Hutcheon@meei.harvard.edu (A.E.K.H.); Xiaoqing_Guo@meei.harvard.edu (X.G.); 2 Department of Ophthalmology/Dean McGee Eye Institute, University of Oklahoma Health Science Center, Oklahoma City, OK 73104, USA; Dimitrios-Karamichos@ouhsc.edu; 3 Department of Cell Biology, University of Oklahoma Health Sciences Center, Oklahoma City, OK 73104, USA

**Keywords:** cornea, extracellular vesicles, co-cultures, epithelium, fibroblasts, stroma, collagen

## Abstract

Cell–cell communication plays a fundamental role in mediating corneal wound healing following injury or infection. Depending on the severity of the wound, regeneration of the cornea and the propensity for scar development are influenced by the acute resolution of the pro-fibrotic response mediated by closure of the wound via cellular and tissue contraction. Damage of the corneal epithelium, basement membrane, and anterior stroma following a superficial keratectomy is known to lead to significant provisional matrix deposition, including secretion of fibronectin and thrombospondin-1, as well as development of a corneal scar. In addition, corneal wounding has previously been shown to promote release of extracellular vesicles from the corneal epithelium, which, in addition to soluble factors, may play a role in promoting tissue regeneration. In this study, we report the development and characterization of a co-culture system of human corneal epithelial cells and corneal stromal fibroblasts cultured for 4 weeks to allow extracellular matrix deposition and tissue maturation. The secretion of provisional matrix components, as well as small and large extracellular vesicles, was apparent within the constructs, suggesting cell–cell communication between epithelial and stromal cell populations. Laminin-1β was highly expressed by the corneal epithelial layer with the presence of notable patches of basement membrane identified by transmission electron microscopy. Interestingly, we identified expression of collagen type III, fibronectin, and thrombospondin-1 along the epithelial–stromal interface similar to observations seen in vivo following a keratectomy, as well as expression of the myofibroblast marker, α-smooth muscle actin, within the stroma. Our results suggest that this corneal epithelial–stromal model may be useful in the study of the biochemical phenomena that occur during corneal wound healing.

## 1. Introduction

The cornea serves as a transparent barrier to protect the eye against environmental stresses. The optical properties of the tissue require high transparency mediated by intricate organization of the stromal extracellular matrix (ECM). Prolonged or irregular wound healing may promote opacification of the cornea leading to fibrotic ECM deposition and ultimately vision loss. Over six million people are visually impaired due to corneal scarring, primarily a result of trachoma infections [1]. Corneal transplantation remains the only therapeutic option to recover vision loss due to corneal scarring. Thus, physiologically relevant models to examine the pharmacological and toxicological effects of infection, chemical application, and disease are needed to develop novel therapeutics to prevent permanent corneal scarring.

Cell–cell communication between the epithelial and stromal layers within the cornea directly influences whether a corneal scar develops. The basement membrane is thought to play a determinant role in stromal fibrosis by regulating the diffusion of soluble, free-factors, such as transforming growth factor-β1 or thrombospondin-1, from the epithelium to the stroma [2,3]. Moreover, we have previously found that the wounded corneal epithelium in vivo secretes extracellular vesicles in response to epithelial debridement that also appear to be restricted by the basement membrane [4]; however, in the absence of a basement membrane, as with a keratectomy, these extracellular vesicles appear to freely disperse into the corneal stroma [4]. We hypothesize that extracellular vesicles may play an important role in corneal regeneration and scar development.

A number of corneal in vitro constructs have been proposed over the years as potential models to study corneal biology. Full-thickness cornea models containing epithelial, stromal, and endothelial cell types, have been constructed by our lab [5], as well as others [6,7], using a collagen hydrogel seeded with corneal fibroblasts and epithelial and endothelial cell layers at the anterior and posterior surfaces, respectively. Co-cultures of corneal epithelial cells and fibroblasts have also been reported with inclusion of amniotic membrane [8], epithelial explants [9], or acellular porcine matrices [10]. A self-assembled approach has also been proposed using stacked ECM sheets generated by human corneal fibroblasts with epithelial cells seeded on top [11]. Other approaches using compressed collagen hydrogels containing stromal fibroblasts with improved biomechanical and optical properties have likewise been reported [12,13]. These models highlight the importance and potential application of multi-cellular systems to study physiological processes in vitro [14].

During corneal wound repair, the corneal epithelium can presumably stimulate fibroblast fibrosis or scarless regeneration. The healing process is thought to be partly regulated by the epithelial–stromal interactions [15]. In this study, we applied an in vitro approach to examine the interaction between the epithelium and a self-secreted fibroblast matrix. The goal of the study was to develop an in vitro co-culture model to examine epithelial–stromal interactions mimicking the wound-healing process. The co-culture system utilizes primary or immortalized human corneal epithelial cells as the epithelial cell layer and self-assembled constructs generated by human corneal fibroblasts (hCFs) as the stromal layer. Primary hCFs have been commonly applied in the development of corneal stromal models [16,17,18]. These cells are derived from keratocytes present in the corneal stroma that become activated upon exposure to serum [19,20]. This model implements a developmental tissue-engineering approach [21] that relies upon stimulated ECM deposition to form an aligned collagenous matrix [17] reminiscent of the developing cornea during embryogenesis (reviewed in [22]). This methodology differs greatly from a classical tissue engineering approach that involves seeding cells within pre-formed scaffolds, and instead relies on in situ collagen production within a quiescent microenvironment in the context of cell phenotype and ECM composition and organization, which is in sharp contrast to the disorganized ECM deposition in the corneal stroma during wound healing [16]. The stromal constructs utilized in this study are formed over 3–4 weeks by promoting resident corneal fibroblasts to secrete and assemble an ECM rich in collagen types I and V mediated by stimulation with a stable Vitamin C derivative [16,17]. Addition of an overlaying epithelial layer in this co-culture system allows for cell–cell interactions between the epithelial and stromal layers. In this study, we evaluated the microscopic structure and cellular components contained within this novel epithelial–stromal co-culture construct, as a potential means to study corneal epithelial–stromal interactions in vitro.

## 2. Materials and Methods

### 2.1. Cell Culture

This study adheres to the Declaration of Helsinki and all donor eyes were received from the National Disease Research Interchange (NDRI, Philadelphia, PA, USA). No identifying information was provided. The institutional review board at the Schepens Eye Research Institute deemed these experiments to be exempt.

#### 2.1.1. Human Corneal Fibroblasts 

Primary hCFs were isolated from corneal tissue and cultured according to established protocols [16,17]. Briefly, following epithelial and endothelial layer debridement, the stroma was cut into small pieces (~2 mm^2^) using a surgical scalpel and allowed to adhere onto the bottom of a T25 flask followed by incubation at 37 °C/5% CO_2_ in complete fibroblast media (10% fetal bovine serum (FBS: Atlanta Biologicals, Flowery Branch, GA, USA), Eagle’s Minimum Essential Medium (EMEM: ATCC, Manassas, VA, USA), and 1X antibiotic-antimycotic (Gibco, Grand Island, NY, USA)) for 2–4 weeks until ~75% confluent [16,17].

#### 2.1.2. Human Corneal Epithelial Cells

Two different human corneal epithelial cell sources were used in these studies and isolated per previously described methods: (1) primary human corneal epithelial cells (hCECs) [23], and (2) the SV40-immortalized epithelial cell line (hCE-TJ) [24,25]. Corneal epithelial cells were sub-cultured in complete epithelial growth media (Keratinocyte-SFM (Gibco), 0.05 mg/mL bovine pituitary extract (Gibco), and 5 ng/mL epithelial growth factor (Gibco)) in T75 flasks coated with fibronectin (Athena Environmental Sciences, Baltimore, MD, USA) at 37 °C/5% CO_2_ until 90% confluent [24,25].

#### 2.1.3. Three-Dimensional (3D) Corneal Stromal Constructs

Stromal constructs generated by hCFs were cultured in 3D model conditions, as previously described [16,17]. Briefly, hCFs (passages 2–4) were seeded into 24 mm polycarbonate transwell plates with 0.4 μm pores (Corning, Corning, NY, USA) at a density of 10^6^ cells/well maintained in complete fibroblast media. At 24 h post-seeding, fresh complete fibroblast media containing a stable Vitamin C (VitC) derivative (2-O-α-D-glucopyranosyl-L-ascorbic acid (CAS # 129499-78-1): Wako Chemicals, Richmond, VA, USA) at a final concentration of 0.5 mM, was added to each well to stimulate ECM production [16,17]. Constructs were maintained in VitC-supplemented complete fibroblast media for 3 weeks for co-culture studies and a total of 4 weeks for monoculture studies with media changes 3× per week. 

#### 2.1.4. Co-Culture of Corneal Epithelium and Stromal Fibroblasts 

To generate corneal construct co-cultures, we seeded hCECs or hCE-TJs (passage 40+) at a density of 10^6^ cells/well on top of a 3-week stromal construct. Complete epithelial and fibroblast media were supplemented in the top and bottom well, respectively, for 4 days and cultured at 37 °C/5% CO_2_. After 4 days, the epithelial media on top of the co-culture was reduced so that only enough media was present to keep the cells moist, thus creating an air-liquid interface. The co-cultures were maintained in this manner for 3–6 additional days. 

### 2.2. TEM

The constructs were collected, fixed in 1/2 strength Karnovsky’s fixative (2% paraformaldehyde and 2.5% gluteraldehyde in 0.1M cacodylate buffer, pH 7.4) [26], and processed for TEM using standard procedures based on established methods [17,27]. After fixation, the constructs were washed in phosphate buffered saline (PBS), post-fixed in 2% osmium tetroxide in 0.1M cacodylate buffer (pH 7.4), and en bloc stained in 0.5% uranyl oxide. The constructs then were dehydrated in a graded alcohol series followed by propylene oxide and embedded (EMbed 812: Electron Microscopy Sciences; Hatfield, PA, USA). Thin sections (60–90 Å) were cut transverse to the plane of the construct using a diamond knife on an ultramicrotome (LKB; Bromma, Sweden), and viewed and imaged with a transmission electron microscope (Tecnai G2 Spirit, FEI Company, Hillsboro, OR, USA).

### 2.3. Immunohistochemistry

Constructs were isolated and fixed in 4% paraformaldehyde in PBS for 30 min at room temperature to overnight at 4 °C followed by permeabilization in 0.1% Triton-X-100 for 10 min to 1 h at room temperature. Blocking was performed in either 1 or 2% bovine serum albumin (Sigma Aldrich, St. Louis, MO, USA) for 1 h at room temperature, and samples were incubated with primary antibody overnight (in 1% bovine serum albumin) at 4 °C with rocking. The samples were then incubated with the secondary antibody, as well as phalloidin and/or TOPRO-3-iodide overnight at 4 °C with rocking. Commercial antibodies utilized in these studies are listed in Table 1. Samples were mounted onto a slide with mounting medium (Vectashield Mounting Medium for fluorescence, Vector Laboratories, Burlingame, CA, USA) and imaged using a Leica TCS SP2 or SP5 confocal microscope (Leica Microsystems, Bannockburn, IL, USA) under oil-immersion with a 40× objective lens and zoom function. A negative control without the primary antibody was performed to verify specificity.

## 3. Results

**Figure 1:**
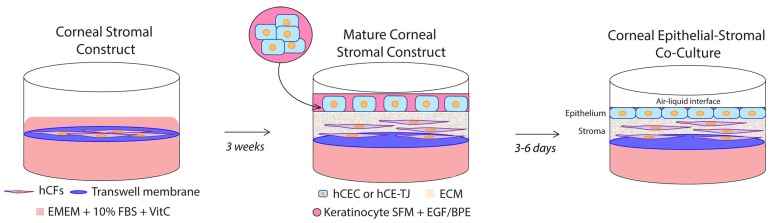
Schematic depicting the assembly of the corneal tissue co-culture system. Human corneal fibroblasts (hCFs) were seeded onto a polycarbonate transwell membrane followed by incubation in 0.5 mM Vitamin C-supplemented complete fibroblast media (Eagle’s Minimum Essential Medium (EMEM) + 10% fetal bovine serum (FBS) + 1X antibiotic/antimycotic) for 3 weeks at 37 °C/5% CO_2_ to allow in situ extracellular matrix (ECM) deposition. Human corneal epithelial cells (hCECs) were then seeded on top of the mature stromal construct and incubated in complete epithelial media (keratinocyte serum-free media (SFM) plus epithelial growth factor (EGF) and bovine pituitary extract (BPE)) for 4 days and airlifted to an air–liquid interface for 3–6 days at 37 °C/5% CO_2_ with complete fibroblast media in the bottom well.

To develop an epithelial–stromal co-culture system, we applied a similar approach to our previous work on the monoculture model [16,17], with the added inclusion of an epithelial layer (Figure 1). Initially, the hCFs were grown on a porous transwell membrane and stimulated to secrete and deposit an ECM, which occurred over 3 weeks in the presence of a stable VitC derivative that is known to stimulate collagen production [28]. The hCECs were then seeded onto the monoculture construct, and the co-culture system was cultivated for an additional 7–10 days, with the hCECs submerged in serum-free epithelial media for 4 days and airlifted to an air–liquid interface for the final 3–6 days, to allow for the maturation of the epithelial layer [5]. The porous transwell system allowed for the inclusion of VitC-supplemented complete corneal fibroblast media within the bottom well and serum-free epithelial media in the top well, thereby permitting specific cellular nutritional support. 

**Figure 2:**
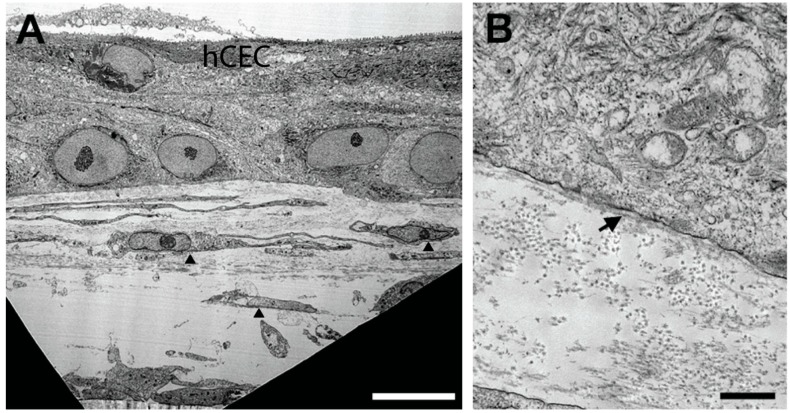
TEM images of the epithelial–stromal constructs. (**A**) The stromal matrix reached a thickness of 30 µm with a total thickness from the epithelial surface to the posterior stroma of 43 µm. The epithelium stratified to 2–3 cell layers with a sparse distribution of hCFs (arrowheads); scale bar = 10 µm. (**B**) High magnification TEM revealed the presence of notable patches of basement membrane (arrow); scale bar = 0.5 µm.

**Figure A1:**
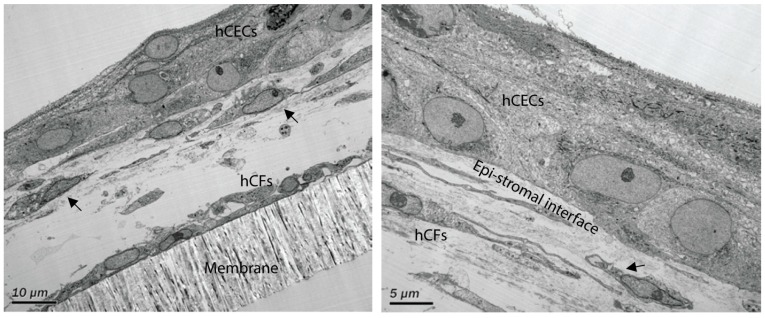
TEM of corneal epithelial–stromal co-cultures at low magnification. 2–3 layers of hCECs formed at the apical side of the construct with dispersion of fibroblasts (arrows) within the self-assembled matrix and on top of the transwell membrane. The total thickness of the construct = 45 μm. Magnification = 1600× (left panel) and 3000× (right panel).

To characterize the cellular and structural morphology of the epithelial–stromal co-culture system, we isolated the constructs and imaged by TEM. We identified minor stratification of hCECs (2–3 layers) with localization of hCFs throughout the construct (Figure 2). Consistent with the sparse distribution of keratocytes within the corneal stroma in vivo [29], the cell abundance of hCFs was higher proximal to the hCEC layer (Figure 2A). Substantial amounts of ECM were seen throughout the construct with a stromal thickness of 27–30 μm and a total thickness of 43–45 μm from the anterior epithelium to the posterior side of the stroma (Figure 2A and Appendix A (Figure A1)). Constructs composed of hCE-TJ/hCFs generated a thinner matrix with total thickness of 21.54 ± 6.58 μm [Appendix A (Table A1)]. The presence of a presumed basement membrane was observed at high magnification oriented along the epithelial–stromal interface (Figure 2B). 

**Figure 3:**
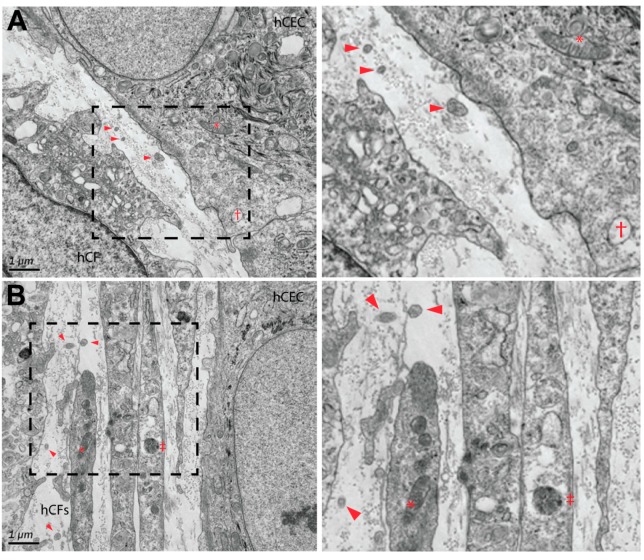
TEM of corneal epithelial–stromal interactions. (**A**) The presence of secreted collagen and extracellular vesicles were apparent between human corneal epithelial cells (hCECs) and human corneal fibroblasts (hCFs) (dashed box enlarged in right panel). (**B**) Extracellular vesicles were also present in between hCF cell populations (dashed box enlarged in right panel). Arrowheads = extracellular vesicles; * = mitochondria; † = vacuole; and ‡ = lysosome. Magnification = 12,000×.

In terms of sub-cellular structure, the abundance of cytoplasmic features in hCECs and hCFs were evident, including mitochondria, lysosomes, and vacuoles (Figure 3). The presence of small vesicles (188 ± 10 nm, range 96–342 nm; arrowheads) was also identified within the extracellular space between hCECs and hCFs (Figure 3A) and between hCFs (Figure 3B). It is likely that these extracellular vesicles may play a fundamental role in cell–cell communication between the epithelium and stromal cell populations [4,30].

**Figure 4:**
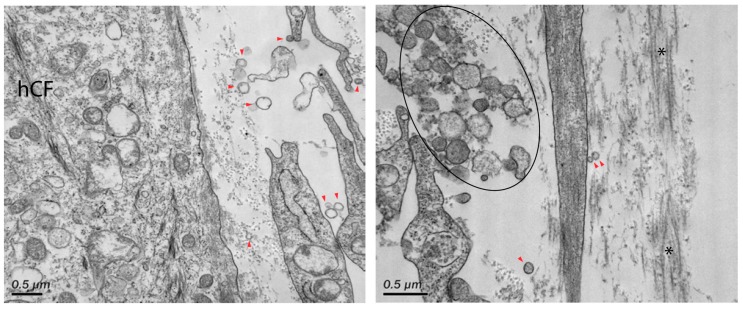
TEM of corneal stromal interactions at high magnification. Secreted extracellular vesicles were identified within the stroma (red arrowheads). Large aggregates of extracellular vesicles (50–330 nm) were also found within the stroma near adjacent cells (black ellipse). Asterisks (*) denote deposited collagen fibrils. Magnification = 21,000× (left panel) and 31,000× (right panel).

The presence of extracellular vesicles (131 ± 5 nm, range 77–228 nm) was also identified between hCFs within the stromal layer of the co-culture constructs (Figure 4, arrowheads). In particular, large vesicular bodies (300–330 nm in diameter) were apparent within the stroma near resident hCFs (Figure 4, right panel ellipse). These vesicles may be attributed as microvesicles, which are larger vesicles (up to 1 μm in diameter) formed directly from the plasma membrane. Some of these large aggregates may also be remnants of apoptotic bodies that have formed as a result of cell turnover within the stroma [31].

**Figure 5:**
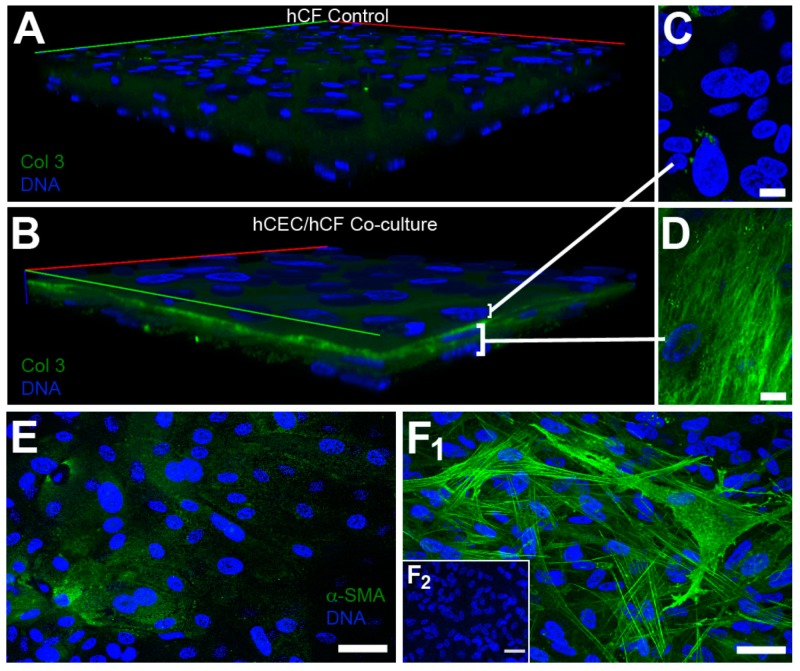
Expression of fibrotic markers in stromal constructs and epithelial–stromal co-cultures. Expression of collagen type III (Col 3) in (**A**) hCF control constructs and (**B**) epithelial–stromal (hCEC/hCF) co-culture. High magnification of a single plane of focus in the (**C**) epithelial layer and (**D**) stromal layer show high expression of collagen type III by hCFs and little expression by hCECs. Expression of α-smooth muscle actin in the (**E**) epithelial layer (max projection) and (**F_1_**) stromal layer (max projection) of a co-culture show similar high expression by hCFs with low expression by hCECs. (**F_2_**) Expression of α-smooth muscle actin in hCF control. Imaged using a 40× objective lens. Scale bar = 10 μm (C and D) and 50 μm (E and F).

To determine if our co-culture model mimicked a stromal injury consistent with the absence of a uniform basement membrane, we stained for fibrotic markers, collagen type III, and α-smooth muscle actin, at 1-week post-epithelial seeding (Figure 5). Collagen type III expression appeared low in hCF monocultures (Figure 5A), but was highly localized at the epithelial–stromal interface in the co-culture system with high expression localized to resident hCFs (Figure 5D). Likewise, we found relatively low expression of α-smooth muscle actin in hCECs (Figure 5E) with high expression by hCFs in epithelial–stromal co-cultures (Figure 5F_1_). Little, if any, α-smooth muscle actin expression was observed in hCF construct only (Figure 5F_2_). The upregulation in α-smooth muscle actin in co-culture suggests that the addition of hCECs promotes differentiation of hCFs to myofibroblasts. 

**Figure 6:**
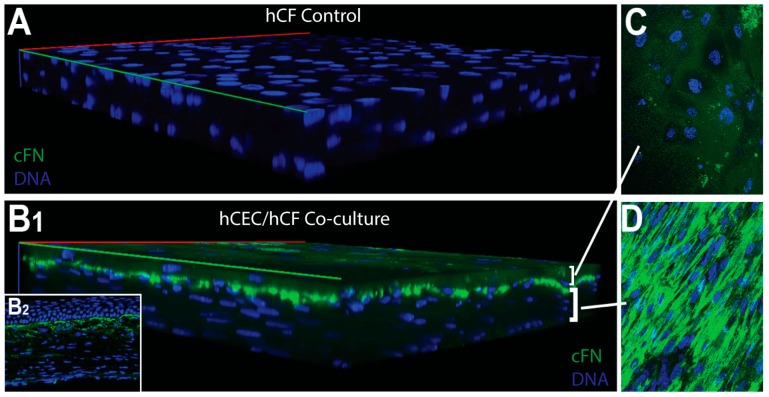
Fibronectin (cFN) expression in hCF stromal constructs and epithelial–stromal co-cultures. (**A**) hCF construct only, showing no fibronectin staining. (**B_1_**) Epithelial–stromal (hCEC/hCF) co-culture showing the expression of fibronectin at the epithelial–stromal interface similar to (**B_2_**) rat cornea 1-week post-keratectomy. (**C**) Max projection image of the hCEC layer showing little expression in the epithelial layer. (**D**) Max projection image of the hCF layer showing high fibronectin expression at the epithelial–stromal interface. Imaged using a 40× objective lens.

**Figure 7:**
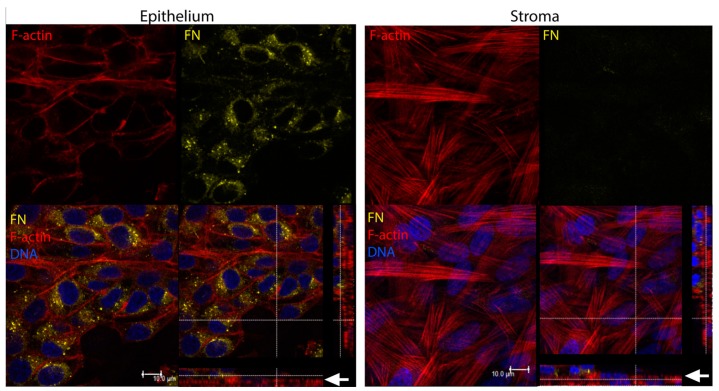
Localization of fibronectin (FN) in corneal epithelial–stromal co-cultures (hCE-TJ/hCF) at 3 days post-airlift. High expression of fibronectin in the epithelial layer (left panel) with little expression in the stromal layer (right panel). Arrows (white) denote the region of the epithelial–stromal interface. Scale bar = 10 μm.

We have previously found that the wounded corneal epithelium in vivo secretes fibronectin into the anterior stroma by 3 days following a keratectomy [32]. Moreover, expression of fibronectin appears to localize at the epithelial–stromal interface and anterior stroma from 3–42 days post-keratectomy [33]. Fibronectin is known to aid in epithelial cell migration post-debridement [34], thus playing an important role in epithelial regeneration. To determine if hCECs express provisional matrix proteins in our co-culture system, we evaluated the expression and localization of fibronectin. We found that control hCFs expressed little to no fibronectin in vitro (Figure 6A). In co-cultures, fibronectin expression appeared to localize to the epithelial–stromal interface, primarily at the anterior side of the stromal layer (Figure 6B–D). This expression pattern was similar to that observed following a keratectomy in vivo [32,35] (Figure 6B_2_). We also examined fibronectin expression in immortalized epithelial cells (hCE-TJ) in 3-day post-airlift co-cultures, and found that hCFs did not appear to express fibronectin; however, the majority of fibronectin was likely derived from the hCE-TJ layer [Figure 7 and Appendix A (Table A2)].

**Figure 8:**
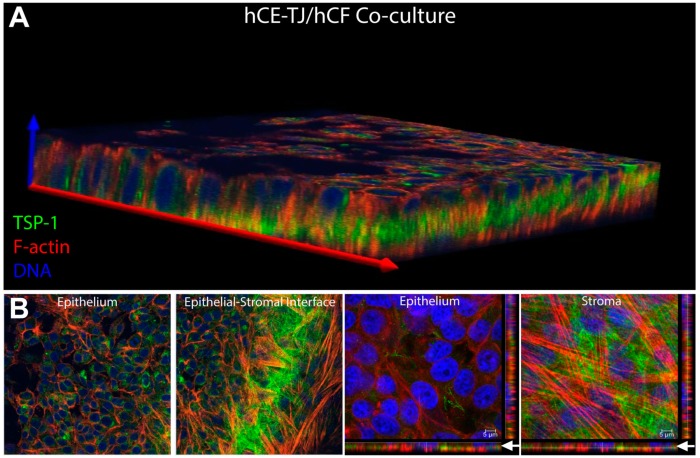
Thrombospondin-1 (TSP-1) expression in corneal epithelial–stromal co-cultures (hCE-TJ/hCF). (**A**) 3D-reconstruction of the corneal co-culture shows localization of thrombospondin-1 primarily at the epithelial–stromal interface. (**B**) Individual slices of the epithelial and stromal layers showed high expression within epithelial cells (left panel) and a fibrous appearance of thrombospondin-1 at the epithelial–stromal interface (right panel). Arrows (white) denote the region of the epithelial–stromal interface.

Given the importance of thrombospondin-1 in corneal wound healing [36], we sought to determine the expression pattern of this ECM protein within the co-culture system. We found that hCE-TJs expressed high amounts of thrombospondin-1 with localization found primarily at the epithelial–stromal interface (Figure 8A), similar to the spatial localization of fibronectin (Figure 6B_1_). Thrombospondin-1 appeared as a fibrous matrix, similar in appearance to collagen, at the apical side of the stromal hCF layer, suggesting that secreted thrombospondin-1 (likely originating from the hCE-TJ-layer) may be binding to stromal collagen deposited by hCFs [Figure 8B and Appendix A (Table A2)]. 

**Figure 9:**
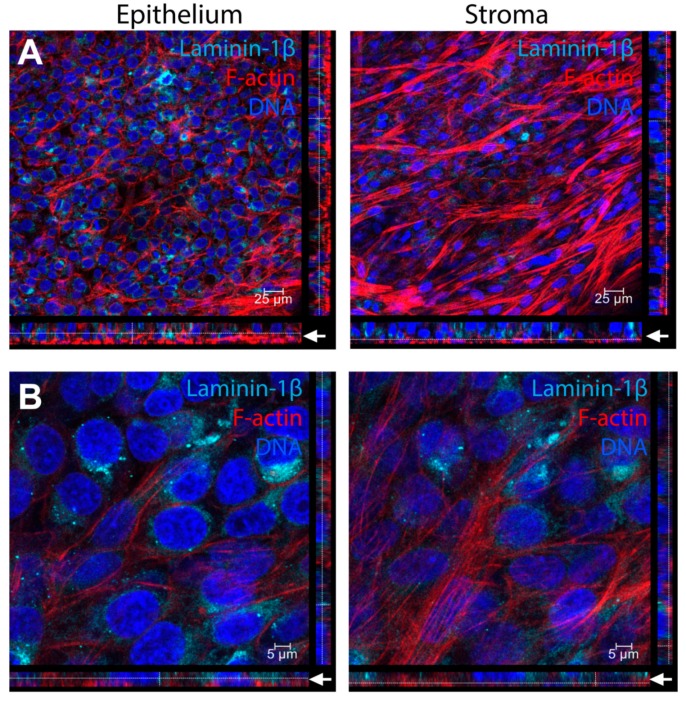
Laminin-1β localization in corneal epithelial–stromal co-cultures (hCE-TJ/hCF) at 3 days post-airlift. Expression of laminin-1β is predominately found within the epithelial layer at the apical side of the hCE-TJ/hCF junction, as can be seen at (**A**) low (40× objective) and (**B**) high (40× objective + zoom 4) magnification. Arrows (white) denote the region of the epithelial–stromal interface. Constructs were isolated at a total incubation period of 4 weeks in culture.

In the human cornea in vivo, an epithelial basement membrane rich in collagen type IV, laminin, heparan sulfate proteoglycans (e.g., perlecan and agrin), and nidogen, separates the epithelial layer from the stromal layer [37,38]. Formation of this basement membrane may play an important role in protecting the stroma from pro-fibrotic factors that are secreted by the epithelium following injury [2,3]. Epithelial cells are considered the dominant source for assembling the epithelial basement membrane with possible contributions from stromal keratocytes [39,40]. To determine if the epithelium begins to express basement membrane proteins by 1 week when cultured in our co-culture system, we stained for laminin-1β, a highly abundant protein found in the epithelial basement membrane [38]. We identified high expression of laminin-1β by hCE-TJs, with localization near the basal surface of the epithelium (Figure 9); however, by 3 days post-airlift, the laminin-1β did not form a cohesive layer at the epithelial–stromal interface (Figure 9A). Little to no expression of laminin-1β was found in hCFs compared to the high expression in hCE-TJs, suggesting that the laminin-1β in this co-culture system was primarily epithelial-derived (Figure 9B). 

## 4. Discussion 

We have previously developed a 3D model of the human corneal stroma that relied upon self-assembly of an ECM produced by hCFs in situ over 4 weeks [16,17]. This 3D stromal model has been applied in the study of endothelial [30] and neuronal [41] cell interactions, as well as in investigations of the effects of hypoxia [42,43] and corneal disease (e.g., keratoconus [44] and diabetes [45]). In this study, we report the development and characterization of corneal epithelial–stromal co-cultures that are constructed using the 3D in vitro model with an overlaying epithelial layer. We found that our corneal co-culture constructs re-capitulated epithelial–stromal interactions during wound healing with clear evidence of extracellular vesicle secretion and deposition of provisional matrix proteins between epithelial and stromal layers. Similar to our findings using endothelial–stromal co-cultures [46], diffusion and uptake of extracellular vesicles within the stroma appeared to occur, suggesting that vesicle migration is not inhibited or limited by the stromal ECM, which is composed predominately of collagen types I and V. The use of this co-culture system as a model of human wound repair has many advantages: (1) it uses human cells and can be easily manipulated to knock-in or -down genes in either the hCEC or hCF; (2) by treating the cultures with EDTA [47], the epithelial layer can be separated from the hCF, allowing for a more precise analysis of both cell groups; and (3) the lack of inclusion of any exogenous scaffold, collagen or synthetic, reduces any interference due to an added substrate and improves the physiological relevance of this system in the study of biological interactions. 

A potential limitation noted in this system is the limited multicellular stratification of the epithelial layer by hCECs and hCE-TJ. The human cornea in vivo contains 5–7 layers of epithelial cell layers, including the superficial epithelial cells, wing cells, and basal cells [48]. While our model showed 1–3 layers of epithelial cells depending on the region of interest, further stratification of the epithelium appeared limited using the epithelial cell line (hCE-TJ) [49]; thus utilization of primary limbal epithelial cells [50] may be required to improve epithelial barrier function. Further validation comparing primary corneal epithelial cells (hCECs) and hCE-TJs is warranted to determine if differences in maturation of the epithelium or barrier function occur depending on the cell type. In addition, we did observe what appeared to be an hCF undergoing apoptosis resulting in the generation of apoptotic bodies within the stroma suggesting cell turnover within the construct. The utilization of hCFs in this model over the quiescent, native keratocyte must also be carefully considered when applying this model in the study of corneal wound healing. While hCFs are easily isolated from the human cornea due to their high proliferative and migratory properties [51], the cellular and morphological properties of hCFs are distinct from the keratocyte with lower expression of the classic keratocyte markers, aldehyde dehydrogenase 3a [52], and keratocan [53]. However, studies have suggested that transfer of hCFs to a serum-free environment may recover the keratocyte phenotype [54].

It is well-established that the wounded corneal epithelium secretes a number of factors, such as transforming growth factor-β1, platelet-derived growth factor, and insulin growth factor, that promote myofibroblast differentiation by stromal keratocytes or fibroblasts that eventually undergo apoptosis [55]. In this study, we found that the addition of hCECs to stromal hCF constructs stimulated a wound-healing response within the hCF population, characterized by high expression of collagen type III and α-smooth muscle actin. The results from our current investigation suggest that this model may be useful in the study of corneal wounding to identify specific free- and bound-factors important in promoting scar development. Of note, the lack of a coherent laminin-1β layer in this co-culture model agrees with previous work, which shows that the corneal endothelium is required for the assembly of a proper epithelial basement membrane [5], suggesting that inclusion of an endothelial layer may be warranted. Whether the corneal endothelium promotes stromal or epithelial expression of basement membrane proteins, or secretes these structural proteins directly for assembly at the anterior surface, remains unclear. 

Further development of in vitro approaches to model the human cornea with additional characterization of the epithelial and stromal interactions during homeostasis and wound healing will likely prove fruitful in developing novel therapeutics to inhibit corneal scar development. Of note, while multiple pathways are involved in corneal scarring and regeneration, targeting the myofibroblast appears to be key to this endeavor [56,57]. The appearance of extracellular vesicles during corneal wound healing [4,46] warrants further study to determine if these membrane-bound factors favor corneal regeneration by increasing matrix deposition and influence myofibroblast persistence. Our results suggest that this co-culture system may be useful in studying epithelial–stromal cell interactions during corneal wound healing. We hypothesize that secretion of provisional matrix components by the corneal epithelium post-injury may be a fundamental mode for promoting tissue closure and regeneration. Further studies are required to characterize the biological features of the developed epithelial–stromal co-culture system to determine if further cultivation of the epithelial layer promotes the downregulation of fibronectin and thrombospondin-1 expression, and the formation of a proper laminin-rich basement membrane.

## Figures and Tables

**Table 1:** Antibodies and labeling agents used for fluorescence microscopy.

Antibody/Labeling Agent	Host Species	Concentration	Manufacturer (City, State; Catalog Number)
Anti-cellular fibronectin (cFN)	Mouse	1:400	Sigma Aldrich (F6140)
Anti-collagen type III	Goat	1:25–1:50	Southern Biotech (Birmingham, AL, USA; 1330-01)
Anti-fibronectin	Goat	1:50	Santa Cruz Biotechnologies (Dallas, TX, USA; SC-90)
Anti-laminin-1β	Rabbit	1:50	Abcam (Cambridge, MA, USA; ab108536)
Anti-α-smooth muscle actin	Mouse	1:25–1:50	Dako North America (Carpinteria, CA, USA; M0851)
Anti-thrombospondin-1	Rabbit	1:50	Abcam (ab85762)
Fluorescein (FITC) AffiniPure Donkey Anti-Goat IgG (H+L)	Donkey	1:100	Jackson ImmunoResearch (Philadelphia, PA, USA; 705-095-147)
Fluorescein (FITC) AffiniPure Donkey Anti-Mouse IgG (H+L)	Donkey	1:100	Jackson ImmunoResearch (715-095-151)
Fluorescein (FITC) AffiniPure Donkey Anti-Mouse IgM	Donkey	1:100	Jackson ImmunoResearch (715-095-140)
Fluorescein (FITC)-phalloidin ^1^	n.a.	1:40	ThermoFisher (Waltham, MA, USA; F432)
Rhodamine (TRITC)-AffiniPure Donkey Anti-Rabbit IgG (H+L)	Donkey	1:100	Jackson ImmunoResearch (711-025-152)
Rhodamine (TRITC)-phalloidin ^1^	n.a.	1:40	Invitrogen (Carlsbad, CA, USA; 1001302)
TOPRO-3-iodide ^2^	n.a.	1:100	Life Technologies (Carlsbad, CA, USA; T-3605)

^1^ Used as a cytoskeletal marker (binds F-actin); ^2^ Used as a nuclear marker (binds DNA).

## Appendix A

**Table A1:** Relative thickness of hCE-TJ/hCF constructs. Based on n = 3 constructs (average ± stdev).

Construct	Thickness
hCE-TJ/hCF co-culture	21.54 ± 6.58 μm

**Table A2:** Relative fluorescence intensity of fibronectin and thrombospondin-1 in the epithelial and stromal regions of hCE-TJ/hCF constructs determined using Fiji [58]. Based on n = 3 constructs (average ± stdev).

Protein	Relative Fluorescence Intensity	
	Epithelium	Stroma
Fibronectin	1346.99 ± 228.17	812.10 ± 422.80
Thrombospondin-1	1477.24 ± 484.14	1200.52 ± 600.91
